# Molecular cloning of rhodanese gene from soil metagenome of cold desert of North-West Himalayas: sequence and structural features of the rhodanese enzyme

**DOI:** 10.1007/s13205-014-0249-2

**Published:** 2014-10-02

**Authors:** Archana Bhat, Syed Riyaz-Ul-Hassan, Nidhi Srivastava, Sarojini Johri

**Affiliations:** 1 Microbial Biotechnology Division, CSIR-Indian Institute of Integrative Medicine (CSIR), Canal Road, Jammu Tawi, 180 001 India; 2 Department of Biosciences and Biotechnology, Banasthali University, Jaipur, India

**Keywords:** Sulfurtransferase, Cyanide, Metagenomic, Cloning, Homology

## Abstract

Rhodanese is a multifunctional, sulfur transferase that catalyzes the detoxification of cyanide by sulphuration in a double displacement (ping pong) mechanistic reaction. In the present study, small-insert metagenomic library from soil sample collected from Ladakh (3,000–3,600 m.a.s.l) in northwestern Himalayas, India was constructed. Function-driven screening of ~8,500 colonies led to the isolation of one esterase-positive clone (clone-est) harboring 2.43 kb insert. Sequence analysis of the insert identified two ORF’s, *phos*M encoding phosphoesterase and *rod*M encoding rhodanese. The 800 bp *rod*M gene encoded a polypeptide of 227 amino acids (RodM). The RodM showed maximum homology with the rhodanese-like protein from *Cyanobacterium synechococcus* species with a score identity of only 51 %. Putative 3D structure of RodM developed by homology modeling resembles to homodimeric protein of SUD sulfur transferase of *Wolinella* *succinogenes* with properly structured active-site cysteine (Cys) residue. Rhodanese has been reported from few culturable microorganisms.

## Introduction

Cyanide is one of the major environmental pollutants produced during certain processes of the chemical and metallurgical industries like steel, electroplating, mining and chemical synthesis. Being extremely toxic to aerobic forms of life, it tightly binds to cytochrome oxidase thereby, inhibiting respiration (Solomonson 1981). Several physical (dilution, membranes, electro wining and hydrolysis/distillation) and chemical methods (alkaline chlorination, ozonation, wet-air oxidation and sulfur based technologies) are being used for the treatment of the cyanide containing wastes but each of these technologies has a relatively high cost and challenges the environment for the release of chemical agents potentially causing secondary pollution (Akcil and Mudder 2003). Bioremediation has been a possible alternative for detoxification of cyanide compounds, and various microbial systems allowing cyanide degradation have been described (Cipollone et al. 2004).

The Thiosulfate:cyanide sulfurtransferases (TST) or rhodaneses (E.C. 2.8.1.1), catalyze the transfer of a sulfane sulfur atom from sulfate to cyanide; using 3-mercaptopyruvate as a sulfur donor (Westley 1973), thus converting to less toxic cyanides, according to the reaction

$${\text{S}}_{2} 0_{3}^{{2 - }} + {\text{ CN}}^{ - } \,\xrightarrow{{{\mathbf{rhodanese}}}}\,{\text{SCN}}^{ - } + {\text{S}}0_{{3 - }}^{2} .$$

Although rhodaneses/sulfurtransferases are widely being distributed in plants and animals but microbes are regarded as the one having efficient mechanisms evolved for cyanide detoxification (Raybuck 1992). Rhodanese has been generally grouped into four categories. Single domain rhodanese TST (GlpE) of 108 amino acids have been demonstrated based on the structural and functional studies of *Escherichia coli* (Ray et al. 2000). Double domain TST’s have been characterized from *Pseudomonas aeruginosa* (Cipollone et al. 2004), *Wolinella* *succinogenes* (Lin et al. 2004) which have been found to consist of two structurally similar domains, each one characterized by a RHOD module containing either a catalytic (Cys) or an inactive aspartic acid (Asp) residue (Bordo and Bork 2002). Multidomain rhodanese-like proteins (ThiI and ThiF/MoeB) involved in the metabolism of the sulfur containing biomolecules (thiamin and molybdopterin) have been characterized (Palenchar et al. 2000). Fourth group of rhodaneses are elongated active-site loop proteins like cdc25 phosphatase which is based on conserved residues in the active-site loop have also been characterized from *Arabidopsis thaliana* (Landrieu et al. 2004). Rhodaneses have been demonstrated and purified from bovine and bacterial sources including *E. coli* (Spallarossa et al. 2004), *Azotobacter vinelandii* (Colnaghi et al. 1996), *Mycobacterium tuberculosis* (Sarah et al. 2008) and *P. aeruginosa* (Cipollone et al. 2004).

Metagenomics, developed over the past decade has been used to identify wide range of potential enzymes from uncultured microorganisms from various environments (Schloss and Handelsman 2003; Streit et al. 2004; Ranjan et al. 2005; Steele and Streit 2005; Rhee et al. 2005). Furthermore, many of these enzymes are found to offer a good potential as new tools for industrial applications.

Ladakh, a region of northwestern Himalayas represents one among such untouched places for the search of new and novel enzymatic activities relevant to biotechnological and industrial applications. Rhodaneses have been isolated from a number of cultivable microorganisms, still many of them remain unknown from uncultivable microbes and need to be explored and exploited. Here we report molecular cloning of the novel gene encoding rhodanese-like thiosulfate sulfur transferase from Ladakh soil metagenome showing 51 % sequence identity at amino acid level with the putative sulfurtransferase from *Synechococcus* sp. PCC 7502. Furthermore, the sequence and structural features of rhodanese-related sulfurtransferase from soil metagenome are discussed.

## Materials and methods

### Bacterial strains, plasmids, and growth conditions

*Escherichia coli* strains JM110 and DH5α were procured from Stratagene (USA), while pUC19 was purchased from (Promega). *E. coli* was grown at 37 °C on Luria–Bertani (LB) medium supplemented with appropriate antibiotics (Sambrook and Russell 2001).

### Collection of environmental sample from Ladakh

Soil samples were collected from Ladakh (34°16′42″North, 77°36′12′15′1.8″East), a desiccated, oligotrophic region at an altitude of 3,000–3,600 m.a.s.l in northwestern Himalayas, India. Soil samples were collected from three dry mineral soil sites (1) LPN1-underneath the snow (2) LPN2-the mid-slopes and (3) LPN3 fine gravels. All the samples were recovered under aseptic conditions by removal of a 200–400 m surface layer of mineral soil from a 20 × 20 cm sample area. These samples were stored in icebox to prevent direct sunlight until processed. All the samples were immediately transported in sterile bottles, polythene bags, etc., and stored at −20 °C until processed. The samples were passed through 2 mm mesh and all the visible roots, rock particles and debris present in the sample were aseptically removed.

### Soil analysis

#### Dry weight assessment and water content

Dry weights were determined by placing 10 g of soil sample (triplicates), in pre-weighed glass petri dishes. The samples were incubated at 100 °C and weighed every 24 h for a period of 3 days using a Mettler-Toledo PE 360 balance. Water content was determined, as the total difference in soil sample weight, expressed as in percentage.

### Construction of metagenomic library

DNA extraction from soil microflora was performed using both manual methods (Direct Lysis and enzymatic methods) (Stach et al. 2001; Miller et al. 1999) and commercial kits (UltraCleanTM Soil DNA isolation Kit (MoBio laboratories/Inc) and FastDNA spin kit (Q-Biogene). Quantification of the DNA was determined using a Nanodrop^®^ ND-100. DNA quality was assessed by the OD ratios at 260/280 nm and analyzed on 0.7 % agarose gel. Standard procedures for molecular cloning were used as described by Sambrook and Russell (2001).

The isolated DNA was partially digested with *Sau*3A1 (Takara) and size fractionated on 0.7 % agarose gel. Standardization of the partial digestion of DNA with *Sau*3A1 was performed first at analytical scale to determine the optimum conditions (time and enzyme dosage) for restriction digestion. The digestion of total metagenomic DNA was carried out at different time intervals i.e., 2′, 4′, 6′, 8′, and 15′ at 37 °C and DNA fragments in the region of 2–10 kb were carefully gel sliced and eluted using gel extraction kit (Qiagen) and purified DNA was visualized on 0.7 % agarose gel. Ligation of the purified, gel eluted 2–10 kb DNA fragments with *Bam*H1 digested pUC19 and further polished with calf intestinal alkaline phosphatase (CIAP) was carried out using T4 DNA Ligase (Fermentas) at 4 °C overnight in a refrigerator. Transformation of ligated mixture (1.5 µl) was performed in DH5α electrocompetent cells using electroporation method. To verify the cloning, transformation mixture was spread on AXI (ampicillin, X-gal, IPTG) plates and incubated at 37 °C overnight. The appearance of blue/white selection of recombinant colonies on plates was seen next day. Recombinant colonies (~20 nos) were selected randomly for the isolation of plasmid followed by digestion with *Bam*H1 to determine the average insert size. The individual ampicillin-resistant plasmid colonies were replicated in 96 well microtiter plates containing 100 μl of LB with ampicillin (75 µg/ml) in each well and incubated at 37 °C overnight. About 100 μl of autoclaved glycerol was added to each well and individual copies of the library were stored at −80 °C.

### Functional screening of metagenomic library for esterase activity

Screening of the metagenomic library for esterase-positive transformants was carried out. The transformants were replicated on LB-agar plates containing 1 % Tributyrin and 0.1 % Tween 80 (HiMedia) with ampicillin (75 µg/ml) and IPTG (30 µM). Plates were incubated for 3 days at 37 °C and regularly checked for zone of hydrolysis. Esterase-expressing colonies surrounded by a clear halo against a creamy background were identified and isolated. The confirmation of the esterase-positive phenotype was performed by plasmid isolation and its restriction analysis. The sequence determination, was performed by fluorescence-based dideoxy DNA cycle sequencing method using Taq Dye Deoxy TM Cycle Sequencing Kit (Applied Biosystems) and with a 310 prism Sequencing System (Applied Biosystems) according to the manufacturer’s instructions.

### Computational analysis

Nucleotide sequence of *rod*M was achieved by submitting the sequence in GenBank database using blastn tool (Altschul et al. 1997). Open reading frame (ORF’s) of the sequence of the inserts were analyzed using ORF finder (http://www.ncbi.nlm.nih.gov/gorf/gorf.html). Signal sequence of the ORF’s were predicted using online server SignalP (http://www.cbs.dtu.dk/services/SignalP). The theoretical molecular mass and isoelectric point (pI) of the amino acids were predicted using ExPASy-ProtParam tool. Homology searches were carried out using GenBank database using BLASTn and BLASTp algorithms (http://www.ncbi.nlm.nih.gov/BLAST). Phylogenetic analysis was performed using DNASTAR programme version 4.0 (Saitou and Nei 1987; Tamura et al. 2007). The nucleotide sequence of *rod*M was deposited in the GenBank database using BankIt programme (info@ncbi.nlm.nih.gov) with an accession number of ADD12003.1.

### Secondary structure analysis and 3D comparative modeling

Comparative alignment of the RodM sequences with the homologous protein sequences downloaded from protein database at NCBI was constructed using ClustalW programme (Thompson et al. 1994). The secondary structure of the sequences was predicted using Sopma method in the Strap program package (http://www.charite.de/bioinf/strap). The sequences were submitted for structure homology analyses using software Phyre2 (http://www.sbg.bio.ic.ac.uk/~phyre/) (Kelley and Sternberg 2009).

## Results

### Collection of soil samples from cold desert of Ladakh

Soil samples from Ladakh region were collected from three different soil layers. Sample LPN1 was fine particulate gravel collected from the upper surface underneath the snow on a slope of 3,600 m.a.s.l of Ladakh region. This soil sample represented a desiccated, oligotrophic habitat, exposed to the harsh environmental conditions. DNA yield from this region after multiple extractions averaged very low (2–5 µg/g of dry soil). Sample LPN2 was recovered between the mid of LPN1 and LPN3 at an altitude of 200 m below the LPN1 site. The sample LPN2 also represented a desiccated and oligotrophic niche with the low DNA yield of 2–6 µg/g of dry soil. The sample LPN3 was an eutrophic soil collected from the underside of the region, 400 m below the LPN2 site. This soil was dark and had a sand-like consistency. LPN3 showed high levels of both protein and lipid and contained almost 8–9 fold more water than the other two sites LPN1 and LPN2. DNA yield from LPN3 was higher (12–16 µg/g of dry soil). LPN3 site was selected for preparation of metagenomic library as this site gave maximum yield of DNA. The properties of all the three soil samples used in this study are listed in Table 1.

**Table 1:** Site descriptions of Ladakh soil samples

Sample code	Site description	Altitude (m)	DNA yield/g soil (µg)	Moisture content (%wt)
LPN1	Dry sand from high altitude site	3,600	2–5	0.65
LPN2	Dry sorted sands and gravels from mid altitude	3,400	2–6	0.8
LPN3	Fine dark particulate soil	3,000	12–16	5.2

### DNA yield and purity

**Fig. 1:**
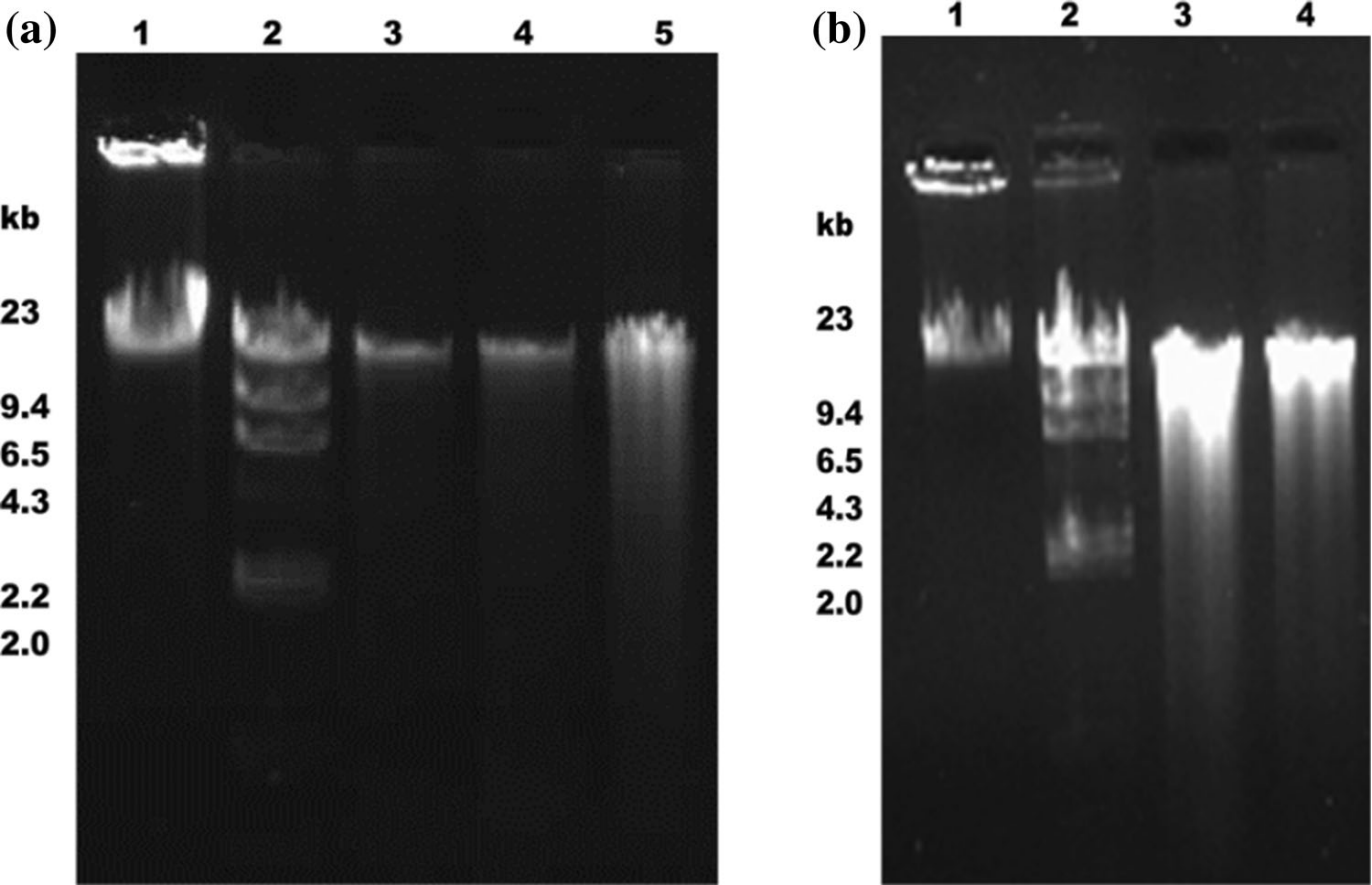
**a** 0.7 % Agarose gel showing DNA isolated from soil using different methods *Lane 1* undigested λ DNA, *Lane 2* λ DNA digested with *Hind* III, *Lane 3* DNA extracted by direct lysis method, *Lane 4* DNA extracted by enzymatic method, *Lane 5* DNA extracted by kit (MoBio). **b** 0.7 % Agarose gel showing DNA extracted from soil using different extraction kits *Lane 1* undigested λ DNA, *Lane 2* λ DNA digested with *Hind* 111, *Lane 3* DNA extracted by MoBio kit, *Lane 4* DNA extracted by Q-Biogene kit

Direct lysis method yielded DNA with a concentration of 8 µg/g of dry soil and a purity index of *A*_260_/*A*_280_ ~0.3 ± 0.6 (Fig. 1a, Lane 3). Soil DNA isolated by enzymatic lysis method was sufficiently pure with purity index of *A*_260_/*A*_280_ ~0.97 ± 0.14, but with almost same yield (7.5 µg/g of dry soil) as that of direct lysis method (Fig. 1a, Lane 4). The Fast DNA spin kit gave DNA of poor quality with purity index of *A*_260_/*A*_280_ ~0.87 ± 0.11 and a yield of <10 µg/g of dry soil (Fig. 1b, Lane 4). However, isolation of soil DNA using MoBio kit yielded high concentration of DNA (16 μg/g of dry soil) with purity index of  *A*_260_/*A*_280_ > 1.8 (Fig. 1b, Lane 2). In addition, the DNA isolated using both the methods was of low molecular weight compared to DNA extracted using kits. High molecular weight metagenomic DNA isolated using MoBio kit with the higher purity index was easily digested using several restriction enzymes like *Mbo*1, *Bam*H1, *Sau3*A1 and *Eco*R1 (data not shown). The *Sau*3A1 digestion resulted in a smearing pattern with the increase in time of incubation, ranging from an intact high molecular weight DNA to ~250 bp. Digested DNA of desired size was optimized for 8′ at 37 °C using 0.01 U/µl *Sau*3A1. The cloning vector pUC19 digested using *Bam*H1 and further ligated with soil DNA gave the appearance of blue/white, selection of colonies on plates.

Using electroporation method, transformation efficiency reached 10^8^ cfu/ml with 96 % of the transformants (4 % tested colonies did not possess insert). A library of approximately ~56,500 colonies was generated; out of these transformants 8,500 colonies were subjected to plate based functional screening. About 15 colonies randomly selected for plasmid isolation showed the presence of plasmid of various sizes inserts ranged between 2 and 5 kb sizes with different restriction patterns which indicated that the library represented a good randomness of the cloned DNA. The average insert size of the DNA was ~4 kb, which covered approximately 226 Mb of total genetic information of Ladakh soil metagenomic library.

### Screening of metagenomic library for esterase activity

Statistically, libraries of 10^7^ transformants need to be screened to ensure a positive hit (Gabor et al. 2004), whereas in this study, metagenomic library constituted only approximately 56,500 transformants which corresponds about <10^5^ transformants. The total amount of genetic information covered in the Ladakh soil metagenomic library was calculated approximately 226 Mb, which is equivalent to 45×, 55×, and 25× the complete genomes of *E. coli* (4.6 Mb), *Bacillus subtilis* (4.3 Mb), and *Streptomyces coelicolor* (9.02 Mb), respectively. Screening of >8,500 transformants colonies on tributyrin plates followed by incubation initially at 37 °C overnight for the growth of colonies and subsequent incubation at 28 °C for 2–4 days for enzyme expression gave five positive hits. One esterase-positive clone which was showing maximum zone of hydrolysis was further selected and designated as clone-est.

### Analysis of nucleotide and amino acid sequence of *rod*M

**Fig. 2:**
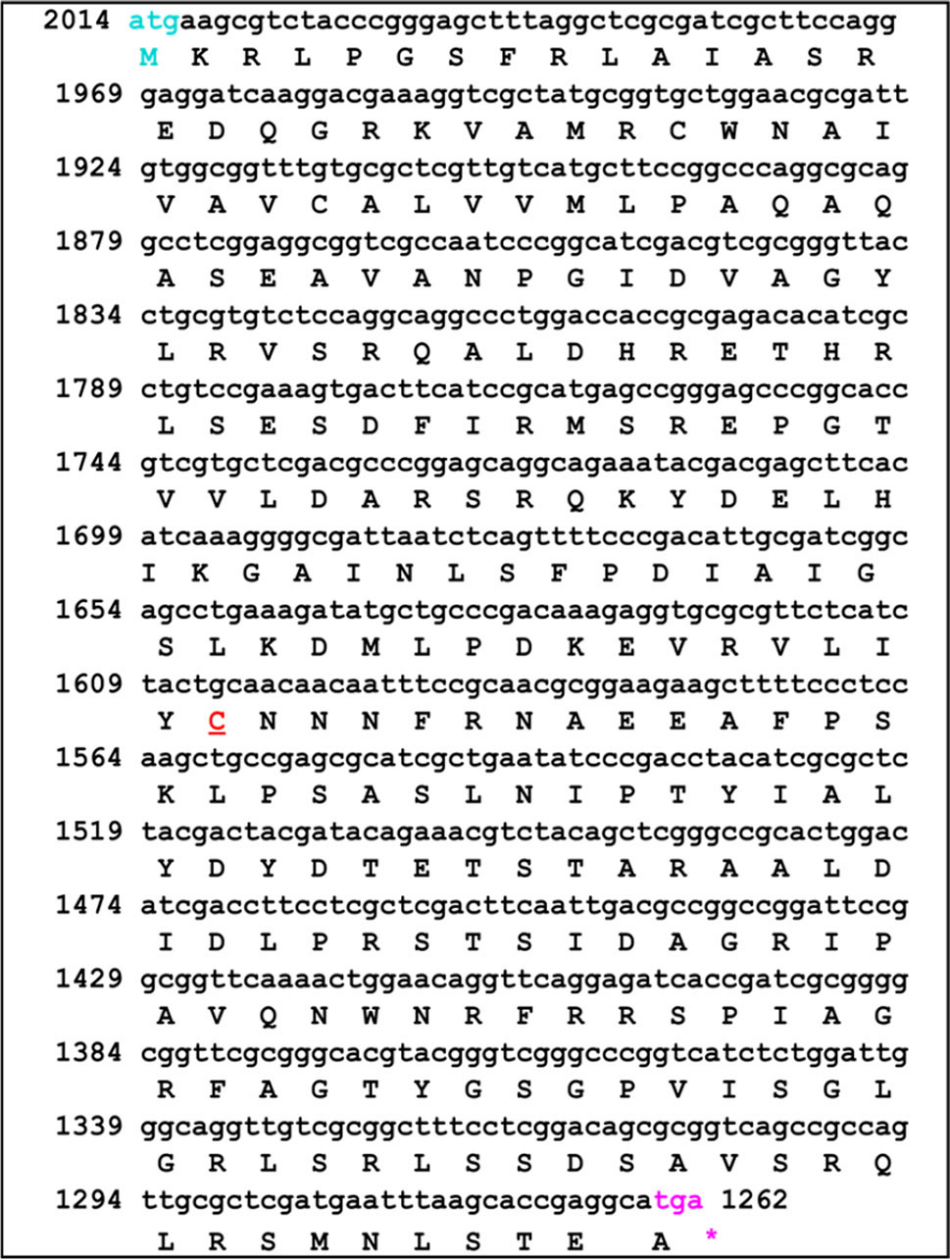
Nucleotide sequence of clone-est showing an ORF of 800 bp

BLASTn and BLASTp sequence analysis of clone-est (2.43 Kb) predicted two different ORF’s. ORF1 (*rod*M) was found to be related to bacterial rhodanase-like proteins and rhodanese-related thiosulfate sulfur transferase and ORF2 (*phosest*M) which was showing similarity with phosphoesterase with 25 % similarity with *Janthinobacterium lividum* (accession number; PAMC 25724). The *rod*M was about 800 bp complete rhodanese gene (Fig. 2). The mature enzyme consisting of 227-residue polypeptide with a predicted molecular mass of 30 kDa and p*I* ~8.8 was predicted by ExPASy-ProtParam.

### Phylogenetic analysis of RodM

**Fig. 3:**
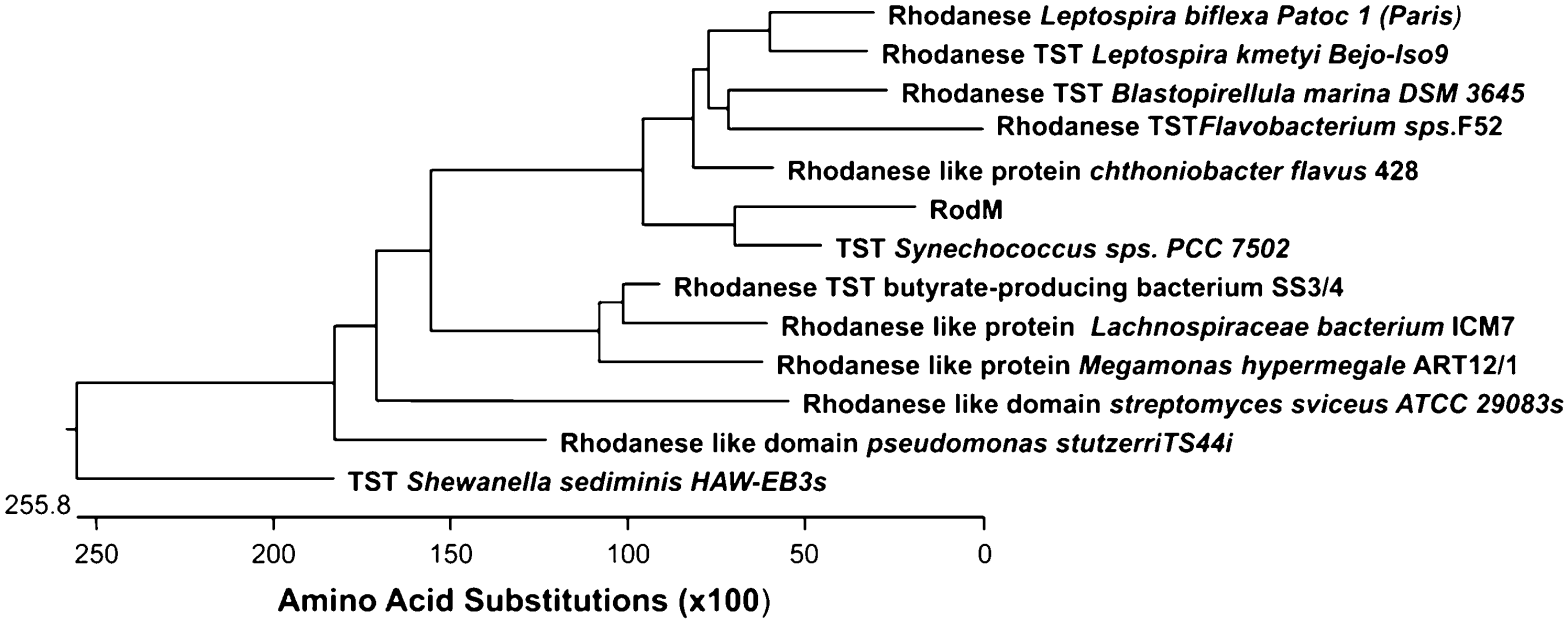
Evolutionary relationship between RodM with closely related bacterial rhodanese families. The tree was constructed according to Tamura et al. (2007)

To classify the protein sequence designated as RodM deduced from *rod*M, a phylogenetic tree was constructed using deduced amino acid sequences from the known rhodanese sequences of diverse sources of microbes as reported in protein database of NCBI. Results suggested that protein RodM was clustered with bacterial rhodanese sequences with putative sulfurtransferase from *Synechococcus* sp. PCC 7502 (GenBank accession no. YP 0071052801) with sequence identity of only 51 % (Fig. 3).

### Secondary structure analysis and 3D comparative modeling

**Fig. 4:**
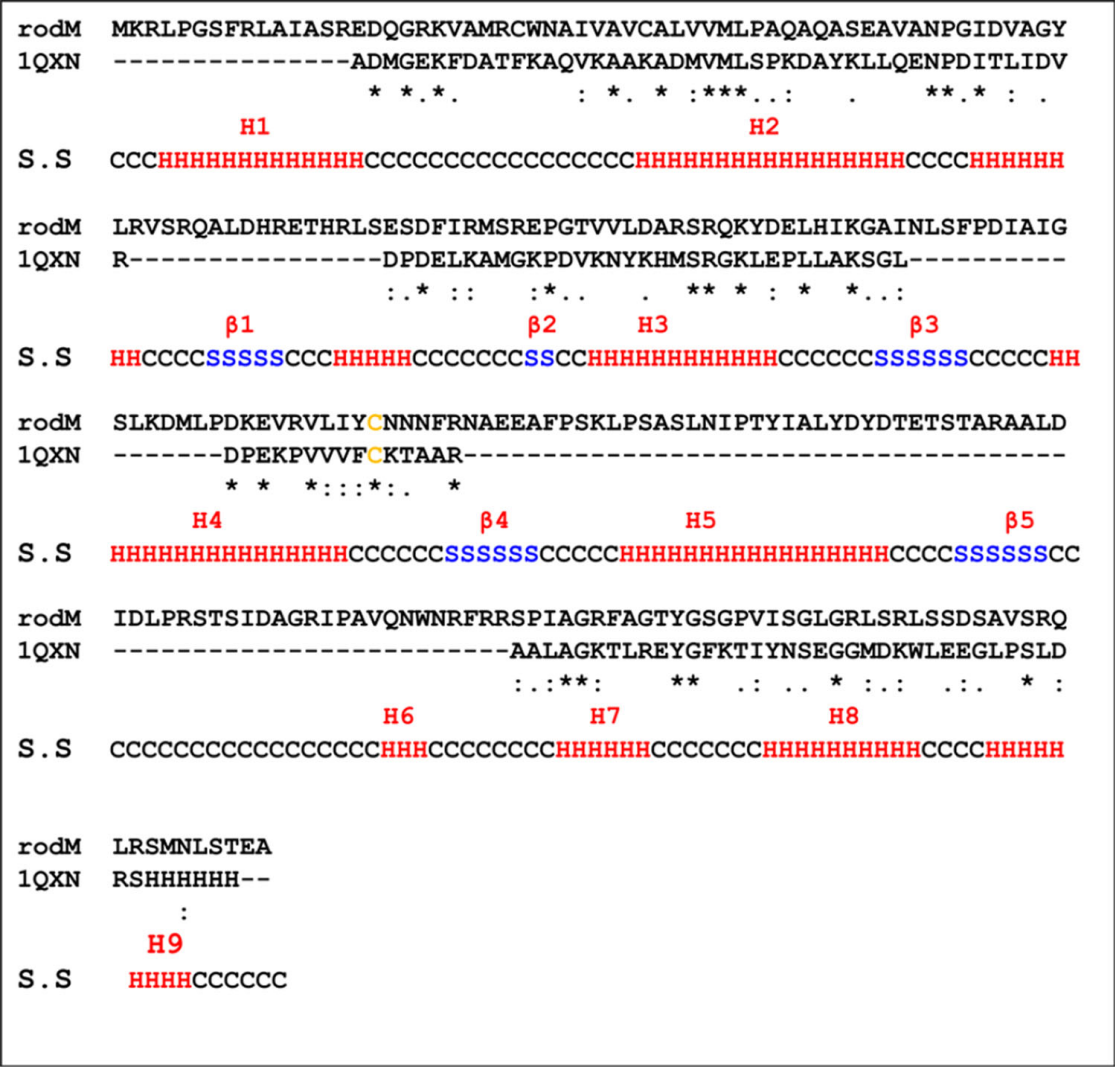
Sequence alignment of RodM (*upper line*) and *Wollinella succinogens* with PDB code 1QXN (*lower line*). Identical residues are indicated by *black asterisks*, non-identical residues in *black dots*, the putative catalytic residues are highlighted in red and conserved domains marked in *green * and *underlined*. Secondary structures are represented by H as α-helices in *red*, S as β-strands in *blue* and C as loops in *black*

ClustalW sequence alignment of RodM showed homology with other sulfur transferases from *E. coli* (PDB ID; 2JTQ) with a score identity of 14.11 %, *Pseudomonas* sp. (PDB ID; C1YT8A) score identity 14.4 %, *Arabidopsis* (PDB ID; At5g66040.1) score identity 10 % (data not shown) and *W.* *succinogenes* (PDB ID; 1QXN) with a score identity of 13.86 %. The sequence alignment of RodM with *W.* *succinogenes* showed similar conserved CXXXXR region with catalytic residue Cys (shown in yellow) at position 114 with nine α-helixes (shown in red) and five β-sheets (shown in blue; Fig. 4).

**Fig. 5:**
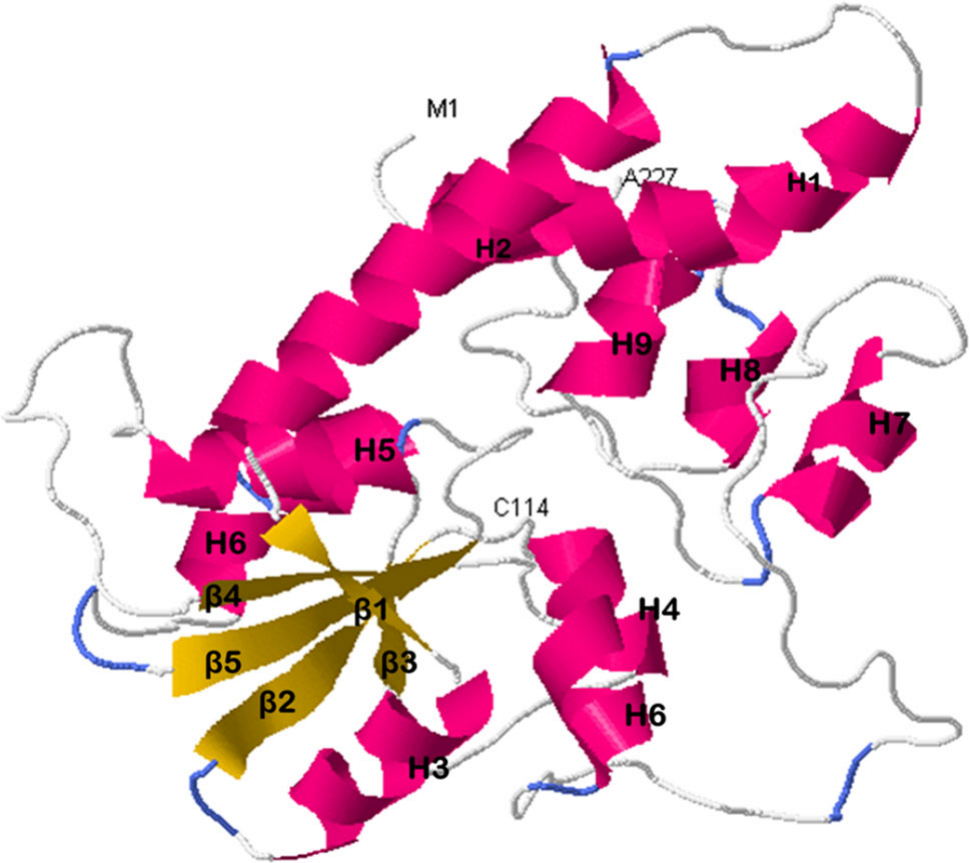
Modeled RodM shown as a cartoon from a top adopts homodimeric barrel structure. Helices are labeled H1–H9 from the N to the C terminus and the β-sheets flanking the substrate binding cleft are shown in *yellow*. The putative catalytic residue Cys are shown at 114 position

Structure predictions of RodM made by the development of the homology model using the resolved X-ray structure of bacterial sulfur transferase (1QXN) from *W.* *succinogenes* as template displayed a typical α/β topology with a heterodimer structure consisting of a five-stranded parallel β-sheet enclosing a hydrophobic core and nine α-helices. The nine helices are labeled as (H1-H9) and the five β-sheets are labeled as (β_1_–β_5_) with N- terminal residue methionene labeled as M1 and C- terminal residue alanine labeled as A227 (Fig. 5). The structure contains a single catalytic active cysteine residue labeled as C114 which may be essential for the tight binding of polysulfide-sulfur and for sulfur transfer as reported in related sulfur transferases (Klimmek et al. 1999).

## Discussion

Rhodanese is sulfur transferase enzyme which is widely distributed in eubacteria, archaea and eukarya (http://smart.emblheidelberg.de/). A substantial proportion of the predicted gene products is functionally uncharacterized or tentatively classified. Analysis of their sequences highlights that they are highly heterogeneous despite the conservation of the rhodanese signatures (Bordo and Bork 2002). Cloning of rhodanese has been reported from a few cultivated microorganisms such as *P. aeruginosa*, *E. coli*, *A. vinelandii* however, isolation of metagenome derived rhodaneses is still lacking. This study is the first report on molecular cloning and identification of rhodanese gene from soil metagenome of cold desert of Ladakh followed by the sequence and structural analysis of rhodanese protein.

In the present study, small-insert soil metagenomic library of cold desert of Ladakh (34°16′42′North, 77°36′15′East), a desiccated, oligotrophic region at an altitude of 3,000–3,600 m.a.s.l in northwestern Himalayas, India was constructed in pUC19 vector. Ladakh, known as cold desert of northwestern Himalaya is unique in many respects for its geo-climatic conditions, like high altitude, extremely cold and dry weather. High altitude northwestern Himalayas has been reported as reservoir of novel and uncultured diversity of microorganisms, from which several novel enzymes have been isolated (Raybuck 1992; Colnaghi et al. 1996). In addition, the region is not exploited by human interference hence it was expected that the soil sample from this area would contain genetic pool encoding novel enzymes that may have industrial application. As soil samples are heterogeneous, details of physical, chemical and biotic factors such as soil type, water content, lipid content, protein content are useful for evaluation and comparison of the outcomes of soil-based studies. Ladakh soil samples (LPN1, LPN2 and LPN3) were analyzed, on the basis of which LPN3 was selected for further study. LPN3 (3,000 m.a.s.l) showed higher levels of both protein and lipid contents (data not shown) and contained almost threefold more water content than LPN1 (3,600 m.a.s.l) and LPN2 (3,400 m.a.s.l).

Soil collection was followed by the isolation and purification of good quantity and quality genomic DNA which represented the microbial community present in the sample. DNA extraction from soil is particularly challenging because it often results in co-extraction of humic substances, which interfere with DNA quantification (Bordo and Bork 2002), inhibit enzymatic manipulations like restriction digestion, PCR amplification, ligation and decreases the transformation efficiency (Cipollone et al. 2004). Several purification procedures for removal of these inhibitory substances have been described, which differ with respect to convenience, quality and yield of DNA (Palenchar et al. 2000; Landrieu et al. 2004; Spallarossa et al. 2004). However, these methods often suffer from incomplete removal of contaminants, are time consuming and laborious, require multiple steps, limit the number of samples or result in significant loss or degradation of DNA (Landrieu et al. 2004; Bordo and Bork 2002). Different methods of DNA isolation have been tried for the preparation of metagenomic library by different workers (Sambrook and Russell 2001). In this study, suitability of both manual methods (direct lysis and enzymatic method) and kit methods (Ultra clean and MoBio, USA) was tested to isolate sufficiently good quality DNA with higher yields. Comparative analysis of DNA isolated using three different techniques indicated that manual methods gave lower yield of DNA (7–8 µg/g soil) with high humic acid content (260/280 ~0.87–0.11) whereas, MoBio kit resulted in sufficiently good quality (16 µg/g soil) and purity DNA (260/280 ~1.7–1.8). DNA isolated using MoBio kit resulted in nearly complete removal of all visible contaminants in one-step, without significant negative impact on DNA quality. DNA isolated by MoBio kit was successfully used for subsequent molecular techniques. In our study, low yields of DNA were obtained from the Ladakh soil in comparison to reports already available in literature on isolation of DNA from various soil samples (Henne et al. 2000). This may be due to the presence of low microbial load in the sandy soil of cold desert of Ladakh. Metagenomic library construction proved successful in this study, as was evident from the average insert size of ~4–5 kb, although the number of colonies generated (56,500) was lower than generated in other studies (Henne et al. 2000; Majernik et al. 2001; Ranjan et al. 2005). This may be due to the several factors such as less diversity of cold environment of Ladakh region. Another factor may be less nucleotide coverage to represent a metagenomic library of Ladakh, etc. The microbial diversity has been reported to be significantly less in very extreme environment compared to a more temperate and less stressed environment such as compost (Sandaa et al. 1999).

Primary sequence analysis of *rod*M showed homology with *Synechococcus* sp. PCC 7502 (GenBank accession no. YP 0071052801) with 51 % similarity at the amino acid level. The structural analysis showed that RodM enzyme shared highest similarity at structural level with SUD from *W. succinogenes.* Other most similar structures of RodM in addition to rhodanese from *W. succinogenes* are from *E. coli* (Alexander and Volini 1987) and *Pseudomonas* sp. (Cipollone et al. 2006). Comparison of the structural domains of RodM with the structures of large size rhodaneses from bovine (Rhobov) or *A. vinelandii* (RhdA) showed some of the common differences at the structural level (data not shown). These differences include shortened loops, less number of α-helices and β-sheets, which result in a convex active-site region in contrast to the corresponding concave areas of Rhobov and RhdA (Spallarossa et al. 2004). The active site cysteine in RodM is located at the central Cys114 position and the starting of the β3-strand of the core five-strand loop, the characteristics of SUD rhodanese (Kreis-Kleinschmidt et al. 1995). Since β-hairpin loop is located on the same side of the protein as the catalytic cysteine loop, its partial mobility suggests that it may play a role in binding of a specific substrate.

Cyanide detoxification by sulfurtransferases/rhodanese has been reported in mammals (Sylvester and Sander 1990), however, *P. aeruginosa* rhodanese is the only one report of cyanide detoxification in prokaryotes (Cipollone et al. 2006). Further, expression and purification studies are being carried out to investigate whether RodM could be used as a tool for the cyanide detoxification.

Our data thus support the possibility of isolating new putative rhodanese gene having low similarity with the already reported rhodaneses from extreme environments of Ladakh using metagenomic approach.
